# Socioeconomic, lifestyle and clinical determinants of health-related quality of life in adults with celiac disease: a national cross-sectional study

**DOI:** 10.3389/fnut.2026.1811468

**Published:** 2026-04-13

**Authors:** Laura Suárez-Barcena González, Julián Rodríguez-Almagro, Alberto Bermejo-Cantarero, Antonio Hernández-Martinez, María Laura Parra-Fernández, Cristina Romero-Blanco

**Affiliations:** 1 Department of Nursing, Physiotherapy and Occupational Therapy, University of Castilla-La Mancha, Ciudad Real, Spain; 2 Instituto de Investigación Sanitaria de Castilla-La Mancha (IDISCAM), Toledo, Spain

**Keywords:** celiac disease, dietary adherence, gluten-free diet, health-related quality of life, socioeconomic factors

## Abstract

**Introduction:**

Health-related quality of life (HRQoL) is a key outcome in the long-term management of celiac disease, yet many adults report persistent limitations despite adherence to a gluten-free diet. Evidence on the independent contribution of socioeconomic, lifestyle, and psychological factors to HRQoL at the population level remains limited.

**Methods:**

We conducted a national cross-sectional study including 1,050 adults with celiac disease living in Spain, recruited through patient associations. Sociodemographic characteristics, lifestyle factors, mental health, and disease-related variables were collected using an online questionnaire. HRQoL was assessed using the disease-specific Celiac Disease Quality of Life questionnaire (CD-QOL), and adherence to a gluten-free diet was measured with the Celiac Dietary Adherence Test (CDAT). Multivariable linear regression models were used to estimate independent associations with overall and domain-specific HRQoL scores.

**Results:**

The mean overall HRQoL score was 59.9 ± 20.1, with the lowest scores observed in the limitations domain. After adjustment, higher income level (>4,000 € vs. < 1,000 €: MD 9.22; 95% CI 3.96–14.48; *p* = 0.001), older age (MD 0.43 per year; 95% CI 0.14–0.71; *p* = 0.003), daily physical activity (MD 4.00; 95% CI 0.43–7.57; *p* = 0.028), and the absence of anxiety (MD 4.90; 95% CI 1.79–8.00; *p* = 0.002) and depression (MD 5.40; 95% CI 0.62–9.45; *p* = 0.025) were independently associated with better HRQoL. In contrast, poorer adherence to a gluten-free diet was consistently associated with worse quality-of-life outcomes across all domains (MD −1.77 per point; 95% CI −2.10 to −1.43; *p* < 0.001). Time since diagnosis and years on a gluten-free diet were not independently associated with HRQoL.

**Discussion:**

These findings indicate that, beyond dietary treatment, HRQoL in adults with celiac disease is primarily shaped by socioeconomic, behavioral, and psychological factors. Population-based strategies that integrate nutritional support with mental health care, physical activity promotion, and measures to reduce socioeconomic barriers may be essential to improve wellbeing and reduce inequalities in this population.

## Introduction

HRQoL is a multidimensional concept that includes the subjective evaluation of physical, psychological, and social functioning, taking into account how illness affects perceived health (1–3). Interest in HRQoL in chronic diseases has increased in recent years, including in digestive diseases, where celiac disease (CD) is a prime example (3–5). However, the available evidence is not uniform. Previous studies differ considerably in their methodological approaches: the populations studied vary in size and geographical origin, the instruments used to assess HRQoL include both generic and disease-specific questionnaires, and the determinants analyzed are not consistent across studies, with some focusing mainly on clinical factors while others incorporate psychological, behavioural or socioeconomic variables (6–9).

CD affects approximately 1%−1.4% of the world's population, can manifest at any age, and is more common in women (1, 10–13). In Spain, the estimated prevalence ranges from approximately 1/71 (about 1.4%) in children to 1/357 (about 0.3%) in adults, suggesting an overall prevalence close to 1% in the general population (14). It is a chronic autoimmune disease characterized by an abnormal response to gluten in genetically susceptible individuals (13, 15). It presents with a heterogeneous clinical picture, including intestinal manifestations (resulting from inflammation and damage to the small intestinal mucosa and villous atrophy) such as abdominal pain, diarrhea, nausea, and vomiting, as well as extraintestinal manifestations that can affect the neurological, dermatological, rheumatological, and gynecological systems, among others (2, 4, 13, 16–20). Furthermore, it is associated with complications such as nutritional deficiencies, osteoporosis, and neoplasms (12, 13, 21).

The diagnosis is based on a combination of compatible clinical presentation, specific serology, and histopathological confirmation via duodenal biopsy; the response to a gluten-free diet (GFD) strengthens the diagnosis (22–24). A GFD is currently the only effective treatment for celiac disease (16, 25). Adherence to this diet improves symptoms and histological alterations (12, 26). Furthermore, several studies have shown that a GFD can bring the HRQoL of people with celiac disease in line with that of the general population or control groups (16, 27, 28). However, adherence to this diet is not always easy, as it depends on factors such as education, professional follow-up, product availability, and cost (12, 13). Recent studies assessing dietary adherence using the Celiac Diet Adherence Test (CDAT) have shown that a considerable proportion of adults with celiac disease experience difficulties maintaining strict adherence to a gluten-free diet, highlighting the importance of monitoring dietary compliance and providing ongoing nutritional support (29). In addition, a high percentage of patients continue to experience symptoms despite good adherence (12). Moreover, HRQoL is part of a biopsychosocial model: it depends not only on the disease itself, but also on individual factors such as mental health, the presence of symptoms, and the time since diagnosis, as well as on community factors such as access to and cost of gluten-free products, professional follow-up, available education, and social support (10, 12, 13, 30). All these elements can act as protective factors or as barriers to wellbeing. In this regard, several studies have indicated that psychological distress (13), the duration of gluten-free diet adherence (27, 28), the presentation of the disease (19), and symptom persistence (12), among other factors, can influence HRQoL.

In Spain, previous studies on HRQoL in celiac disease (CD) exist, but they are less recent and have been based on a limited number of variables, which restricts the overall understanding of its determinants (3, 8, 31). Although factors such as income, physical activity, substance use, and social associations have been previously analyzed, no national study has evaluated them together within a broad framework of variables or using multivariate models that allow for estimating their independent impact. Understanding these determinants is essential to inform follow-up strategies and multidisciplinary care in routine gastroenterology practice.

To update and expand the national evidence, our objective was to analyze the different social and clinical factors associated with HRQoL in adults with celiac disease, both individually and collectively, in a representative sample.

## Methods

### Design and participant selection

A cross-sectional study was conducted in Spanish adults with celiac disease. Participants aged 18 years or older residing in Spain, with a medical diagnosis of celiac disease documented in their medical records, and who provided informed consent were included. The questionnaire was disseminated nationwide through Celiac Disease Associations (e.g., via mailing lists, websites, and social media), enabling broad outreach across different regions of the country. Importantly, participation was open to both association members and non-members, as the survey link was publicly accessible and could be shared beyond the associations' direct networks. Participants who did not meet these criteria or who did not have sufficient Spanish proficiency were excluded. Incomplete questionnaires were also excluded. No additional exclusion criteria were applied regarding the presence of physical or psychological comorbidities. Pregnancy or breastfeeding were also not used as exclusion criteria.

The sample size calculation was based on an estimated prevalence of 1% (14), a 95% confidence level, and an absolute error of 0.6%. The population frame was the population residing in Spain as of January 1, 2024: 48,619,695 inhabitants (32). Based on these parameters, a minimum sample size of 1,056 participants was estimated.

### Information sources

Data collection was carried out using an online questionnaire distributed through celiac disease associations in Spain, which disseminated the survey link among adults with celiac disease through their communication channels. Participation was voluntary and not restricted to association members. The invitation included information about the study, its objectives, an informed consent form, and an email address for inquiries. The questionnaire was developed by the research team based on variables commonly assessed in previous studies on celiac disease and informed by constructs derived from validated reference instruments.

### Study variables

The independent variables included 1) sociodemographic data: sex, age and income; 2) behavioral and health data: frequency of exercise, assessed through a self-reported question and categorized as never or rarely (less than once per week), once per week, several times per week (2–4 times), and daily (≥5 times per week); body mass index (BMI) calculated from self-reported height and weight without specific measurement instructions; physical and psychological comorbidities (including anxiety and depression, based on self-reported prior diagnosis) and tobacco use; and 3) parameters of disease progression: age at diagnosis, years since diagnosis, time on a gluten-free diet, adherence measured with the CDAT, and association membership.

The CDAT is a seven-item questionnaire that evaluates different aspects related to gluten-free diet adherence, including symptoms, self-efficacy, motivation, and potential gluten exposure. It was originally developed by Leffler et al. (33) and later validated in Spanish by Fueyo-Díaz et al. (34). Scores range from 7 to 35; scores < 13 reflect good adherence and >17, limited adherence.

The dependent variable was HRQoL, assessed using the Celiac Disease Quality of Life (CD-QOL) questionnaire. This instrument was developed and validated by Dorn et al. (35) in 2010 and adapted linguistically and culturally to Spanish by Casellas et al. (36) in 2013. It consists of 20 items with five-point Likert-type responses, organized into four dimensions: dysphoria, limitations, health problems, and inadequate treatment. Scores are converted to a scale of 0 to 100, with values of 70 or higher considered indicative of good HRQoL. Following the findings of Fueyo-Díaz et al. (8), and which we also detected during the instrument review, there is a wording discrepancy in item 8: in the original version (35) and, at least, in the Spanish adaptation (36), the statement appears in a positive sense when, for consistency with the “inadequate treatment” subscale and the additive sum of the items, it should be formulated in a negative sense (“I feel that diet is not a sufficient treatment for my illness”). Therefore, in our analyses, we treated item 8 as having a negative meaning.

### Statistical analysis

First, descriptive statistics were performed. Qualitative variables were described as absolute and relative frequencies. Quantitative variables were initially assessed for normality using the Kolmogorov–Smirnov test and, as they showed a normal distribution, they were presented as mean and standard deviation (SD).

Next, bivariate analysis was conducted. Means were compared between categories using Student's t-test or one-way ANOVA, as appropriate. For continuous predictors, linear regression was applied, estimating the change in score per unit of the predictor.

Finally, to estimate independent associations and isolate the specific effect of each predictor, multiple linear regression models were fitted with the overall CD-QOL score and each of its four dimensions as dependent variables. All independent variables described in the study (sex, age, income, physical activity, BMI, physical and psychological comorbidities, tobacco use, age at diagnosis, years since diagnosis, time on a gluten-free diet, CDAT score, and association membership) were included simultaneously in the multivariable models to estimate adjusted mean differences.

Results were expressed as mean differences (MD) with their 95% confidence intervals (95% CI) and *p*-values.

Internal consistency of the CD-QOL and CDAT scales was assessed using Cronbach's alpha coefficient.

A significance level of *p* < 0.05 was used. The analysis was performed using SPSS software, version 29.0.

### Ethical and legal considerations

This study was evaluated and approved by the Social Research Ethics Committee (CEIS, *for its initials in Spanish*) of the University of Castilla La Mancha (Registration No. CEIS-2024-36901).

## Results

The sample consisted of 1,050 participants, whose characteristics are described in Table 1. A total of 923 were women (87.9%), and the mean age was 36.49 ± 11.23 years. In socioeconomic terms, the most frequent income range was €1,000–2,000 per month, with 402 participants (38.3%), followed by €2,000–3,000 per month, with 306 participants (29.1%). Regarding physical activity, 384 (36.6%) exercised several times a week, while 286 (27.2%) reported never or rarely exercising. The mean BMI was 23.37 ± 4.35 kg/m^2^.

**Table 1:** Cohort description.

Variable	*n* (%)	Mean ±SD
Total sample	1,050 (100.0)	
Females	923 (87.9)	
Males	127 (12.1)	
**Age (years)**		36.49 ± 11.23
Income level		
< 1,000 €	84 (8.0)	
1,000–2,000 €	402 (38.3)	
2,000–3,000 €	306 (29.1)	
3,000–4,000 €	166 (15.8)	
>4,000 €	92 (8.8)	
Physical activity		
Never or rarely	286 (27.2)	
Once a week	237 (22.6)	
A few times a week	384 (36.6)	
Every day	143 (13.6)	
BMI (kg/m^2^)^***^		23.37 ± 4.35
Age CD diagnosed (years)^***^		25.97 ± 14.51
Time since CD diagnosis (years)^*^		10.26 ± 9.51
Time on GFD (years)^*^		10.06 ± 9.14
CDAT (7–35)		12.57 ± 3.40
Member of a CD association		
Yes	526 (50.1)	
No	524 (49.9)	
Comorbidities		
Hypertension	42 (4.0)	
Diabetes mellitus	27 (2.6)	
Thyroid disease	125 (11.9)	
Dyslipidemia	84 (8.0)	
Asthma	36 (3.4)	
Depression	94 (9.0)	
Anxiety	214 (20.4)	
Smokers^**^	142 (13.5)	
Cigarettes/day (non-smokers = 0)		1.21 ± 3.67
CD-QOL (0–100)		
Global score		59.90 ± 20.07
Dysphoria		81.07 ± 20.32
Limitations		52.66 ± 24.18
Health problems		58.43 ± 25.94
Inadequate treatment		53.82 ± 24.91

^*^Missing 3 (N: 1,047);

^**^Missing 4 (N: 1,046);

^***^Missing 8 (N: 1,042).

Minimum wage in Spain for 2026: €1,221/month (€17,094/year) (37).

Regarding the clinical history of celiac disease, the mean age at diagnosis was 25.97 ± 14.51 years. The mean time since diagnosis was 10.26 ± 9.51 years, and the mean time on a gluten-free diet was 10.06 ± 9.14 years. Adherence to the diet, as measured by the CDAT, had a mean score of 12.57 ± 3.40 points. Half of the sample (526 participants, 50.1%) reported belonging to a patient association.

The most prevalent comorbidities were anxiety in 214 people (20.4%), thyroid disease in 125 (11.9%), and depression in 94 (9.0%). Dyslipidemia was also reported in 84 participants (8.0%), hypertension in 42 (4.0%), asthma in 36 (3.4%), and diabetes mellitus in 27 (2.6%). Active smoking was reported by 142 people (13.5%), with a mean of 1.21 ± 3.67 cigarettes/day, including non-smokers.

HRQoL assessed using the CD-QOL had a mean score of 59.90 ± 20.07 points. By dimension, mean scores were: dysphoria 81.07 ± 20.32 points, health problems 58.43 ± 25.94 points, limitations 52.66 ± 24.18 points, and inadequate treatment 53.82 ± 24.91 points. The internal consistency of the CD-QOL was high for the overall scale (Cronbach's α = 0.931). For the subscales, α values were 0.813 (Dysphoria), 0.911 (Limitations), 0.869 (Health problems), and 0.312 (Inadequate Treatment). Without reverse-coding Item 8, the α value for the latter subscale was −0.453. The CDAT showed a Cronbach's α of 0.526.

In the bivariate analysis (Table 2), overall quality of life showed better scores among older individuals (*p* < 0.001), those with higher incomes (*p* < 0.001), those who exercised regularly (*p* < 0.001), those without anxiety (*p* < 0.001), those without depression (*p* < 0.001), those with more years since their celiac disease diagnosis (*p* < 0.001), those who had followed a gluten-free diet for more years (*p* < 0.001), and those who belonged to patient associations (*p* < 0.001). In contrast, worse overall scores were observed in those with a later diagnosis (*p* < 0.001), those with suboptimal adherence to a gluten-free diet (*p* < 0.001), and those with a higher BMI (*p* = 0.004).

**Table 2:** Association between sociodemographic and clinical factors bivariable analysis.

Variables	Global score		Dysphoria		Limitations		Health problems		Inadequate treatment	
	aMD (95% CI)	*P*-value	aMD (95% CI)	*P-*value	aMD (95% CI)	*P*-value	aMD (95% CI)	*P*-value	aMD (95% CI)	*P-*value
Gender (Woman)	1.39 (−2.33 to 5.12)	0.463	0.53 (−3.23 to 4.31)	0.780	2.31 (−2.33 to 6.88)	0.297	2.22 (−3.23 to 7.04)	0.365	**–**3.45 (−2.33 to 1.17)	0.143
Age (years)	**0.24** (−2.33 to 0.34)	**<0.001**	**0.14** (−2.33 to 0.25)	**0.007**	**0.34** (−2.33 to 0.46)	**<0.001**	**0.16** (−2.33 to 0.30)	**0.019**	**0.17** (−2.33 to 0.30)	**0.012**
Income 1,000–2,000 € vs. <1,000 €	**6.32** (−2.33 to 10.99)	**0.008**	3.93 (−2.33 to 8.68)	0,104	**6.97** (−2.33 to 12.61)	**0.015**	**6.86** (−2.33 to 12.89)	**0,026**	**6.841** (−2.33 to 12.68)	**0.022**
Income 2,000–3,000 € vs. <1,000 €	**8.56** (−2.33 to 13.77)	**0.001**	**7.41** (−2.33 to 12.72)	**0.006**	**7.84** (−2.33 to 14.13)	**0.015**	**10.01** (−2.33 to 16.74)	**0.004**	**10.49** (−2.33 to 17.00)	**0.002**
Income 3,000–4,000 € vs. <1,000 €	**10.99** (−2.33 to 15.78)	**<0.001**	**8.68** (−2.33 to 13.56)	**<0.001**	**11.11** (−2.33 to 16.90)	**<0.001**	**12.96** (−2.33 to 19.15)	**<0.001**	**10.17** (−2.33 to 16.16)	**0.001**
Income> 4,000 € vs. <1,000 €	**14.17** (−2.33 to 20.04)	**<0.001**	**8.96** (−2.33 to 14.93)	**0.003**	**15.60** (−2.33 to 22.70)	**<0.001**	**18.03** (−2.33 to 25.61)	**<0.001**	**8.51** (−2.33 to 15.86)	**0.023**
Exercise: once/week vs. never or rarely	1.66 (−2.33 to 5.08)	0.341	1.95 (−2.33 to 5.43)	0.272	1.42 (−2.33 to 5.55)	0.498	1.62 (−2.33 to 6.06)	0.474	2.23 (−2.33 to 6.48)	0.303
Exercise: a few times a week/week vs. never or rarely	**5.84** (−2.33 to 8.88)	**<0.001**	**5.53** (−2.33 to 8.63)	**<0.001**	**5.95** (−2.33 to 9.62)	**0.002**	**6.77** (−2.33 to 10.72)	**0.001**	3.65 (**–**012.)	0.058
Exercise: everyday vs. never or rarely	**9.17** (−2.33 to 13.15)	**<0.001**	**5.94** (−2.33 to 10.00)	**0.004**	**10.50** (−2.33 to 15.32)	**<0.001**	**8.21** (−2.33 to 13.39)	**0.002**	**11.97** (−2.33 to 16.93)	**<0.001**
BMI (kg/m^2^)	**−0.41** (−2.33 to −0.13)	**0.004**	**−0.43** (−2.33 to −0.15)	**0.003**	**−0.28** (−2.33 to 0.05)	**0.100**	**−0.65** (−2.33 to −0.29)	**<0.001**	**−0.39** (−2.33 to −0.04)	**0.026**
Age at diagnosis (per year)	**−0.16** (−2.33 to −0.08)	**<0.001**	**−0.21** (−2.33 to −0.12)	**<0.001**	**−0.14** (−2.33 to −0.04)	**0.005**	**−0.17** (−2.33 to −0.06)	**0.002**	**−0.15** (−2.33 to −0.04)	**0.004**
Time since diagnosis (per year)	**0.69** (−2.33 to 0.82)	**<0.001**	**0.69** (−2.33 to 0.81)	**<0.001**	**0.78** (−2.33 to 0.92)	**<0.001**	**0.58** (−2.33 to 0.74)	**<0.001**	**0.62** (−2.33 to 0.77)	**<0.001**
Time on GFD (per year)	**0.74** (−2.33 to 0.87)	**<0.001**	**0.74** (−2.33 to 0.86)	**<0.001**	**0.81** (−2.33 to 0.97)	**<0.001**	**0.65** (−2.33 to 0.82)	**<0.001**	**0.68** (−2.33 to 0.84)	**<0.001**
CDAT score	**−2.31** (−2.33 to −1.98)	**<0.001**	**−2.34** (−2.33 to −2.00)	**<0.001**	**−2.42** (−2.33 to −2.01)	**<0.001**	**−2.20** (−2.33 to −1.76)	**<0.001**	**−2.05** (−2.33 to −1.62)	**<0.001**
Member of a CD association	**5.23** (−2.33 to 7.64)	**<0.001**	**5.38** (−2.33 to 7.82)	**<0.001**	**5.18** (−2.33 to 8.09)	**<0.001**	**4.36** (−2.33 to 7.50)	**0.006**	**7.34** (−2.33 to 10.32)	**<0.001**
Has comorbidity	**–**0.11 (−2.33 to 2.64)	0.933	0.21 (−2.33 to 3.01)	0.882	0.46 (−2.33 to 3.79)	0.784	**–**0.31 (−2.33 to 3.25)	0.863	**–**2.91 (−2.33 to 0.51)	0.096
No anxiety	**11.59** (−2.33 to 14.52)	**<0.001**	**11.18** (−2.33 to 14.16)	**<0.001**	**13.70** (−2.33 to 17.24)	**<0.001**	**9.98** (−2.33 to 13.83)	**<0.001**	**6.92** (−2.33 to 10.65)	**<0.001**
No depression	**15.32** (−2.33 to 19.47)	**<0.001**	**16.67** (−2.33 to 20.86)	**<0.001**	**17.24** (−2.33 to 22.26)	**<0.001**	**12.07** (−2.33 to 17.52)	**<0.001**	**12.08** (−2.33 to 17.32)	**<0.001**
Smoker	**–**1.45 (−2.33 to 2.10)	0.423	0.49 (−2.33 to 4.10)	0.787	**–**0.86 (−2.33 to 3.42)	0.691	**–**2.61 (−2.33 to 1.98)	0.266	**−5.11** (−2.33–−0.70)	**0.023**

MD, mean difference; CI, confidence interval. Bold values indicate statistically significant results (*p* <0.05).

A very similar pattern was observed across all four dimensions of the CD-QOL: higher income, regular exercise, absence of anxiety and depression, more years since diagnosis, longer time on a gluten-free diet, and membership in support groups were consistently associated with better scores. Conversely, suboptimal adherence and a higher BMI were associated with worse outcomes in all dimensions.

No significant associations were observed between sex and physical comorbidity on the overall CD-QOL score or its dimensions. Tobacco use, on the other hand, only showed a negative effect on the inadequate treatment dimension (*p* = 0.023).

In the multivariate analysis (Table 3), overall quality of life was independently associated with higher income, especially the >€4,000 bracket compared to < €1,000 (MD 9.22; 95% CI 3.96 to 14.48); with older age (MD 0.43; 95% CI 0.14 to 0.71); with daily exercise compared to never/rarely (MD 4.00; 95% CI 0.43 to 7.57); and with the absence of anxiety (MD 4.90; 95% CI 1.79 to 8.00) and depression (MD 5.40; 95% CI 0.62 to 9.45). Conversely, poorer adherence to a gluten-free diet was consistently associated with lower overall scores (MD −1.77 per point; 95% CI −2.10 to −1.43).

**Table 3:** Association between sociodemographic and clinical factors multivariate analysis.

Variables	Global score		Dysphoria		Limitations		Health problems		Inadequate treatment	
	aMD (95% CI)	*P*-value	aMD (95% CI)	*P*-value	aMD (95% CI)	*P*-value	aMD (95% CI)	*P*-value	aMD (95% CI)	*P*-value
Gender (woman)	−1.02 (−2.33 to 2.22)	0.536	−1.67 (−2.33 to 1.65)	0.323	−0.35 (−2.33 to 3.68)	0.865	0.31 (−2.33 to 4.90)	0.893	**−6.10** (−2.33 to −1.70)	**0.007**
Age (per year)	**0.43** (−2.33 to 0.71)	**0.003**	0.22 (−2.33 to 0.51)	0.128	**0.52** (−2.33 to 0.87)	**0.003**	**0.56** (−2.33 to 0.96)	**0.006**	0.09 (−2.33 to 0.47)	0.640
Income 1,000–2,000 € vs. <1,000 €	2.10 (−2.33 to 6.22)	0.317	−0.49 (−2.33 to 3.73)	0.818	2.41 (−2.33 to 7.54)	0.355	3.48 (−2.33 to 9.30)	0.240	2.42 (−2.33 to 8.01)	0.395
Income 2,000–3,000 € vs. <1,000 €	3.18 (−2.33 to 7.82)	0.179	1.96 (−2.33 to 6.71)	0.419	2.30 (−2.33 to 8.07)	0.433	5.36 (−2.33 to 11.92)	0.109	4.10 (−2.33 to 10.38)	0.200
Income 3,000–4,000 € vs. <1,000 €	**6.16** (−2.33 to 10.42)	**0.005**	3.62 (−2.33 to 7.99)	0.104	**6.05** (−2.33 to 11.34)	**0.025**	**8.82** (−2.33 to 14.84)	**0.004**	5.14 (−2.33 to 10.91)	0.080
Income >4,000 € vs. <1,000 €	**9.22** (−2.33 to 14.48)	**0.001**	4.05 (−2.33 to 9.45)	0.140	**10.44** (−2.33 to 16.98)	**0.002**	**14.12** (−2.33 to 21.55)	**<0.001**	1.82 (−2.33 to 8.94)	0.616
Exercise: once/week vs. never or rarely	−0.79 (−2.33 to 2.18)	0.599	−0.35 (−2.33 to 2.70)	0.818	−1.13 (−2.33 to 2.57)	0.547	−0.87 (−2.33 to 3.34)	0.685	0.044 (−2.33 to 4.08)	0.983
Exercise: a few times a week/week vs. never or rarely	2.49 (−2.33 to 5.19)	0.069	2.55 (−2.33 to 5.32)	0.070	2.25 (−2.33 to 5.60)	0.188	3.53 (−2.33 to 7.34)	0.069	0.860 (−2.33 to 4.51)	0.644
Exercise: everyday vs. never or rarely	**4.00** (−2.33 to 7.57)	**0.028**	1.38 (−2.33 to 5.04)	0.459	**4.92** (−2.33 to 9.36)	**0.030**	3.01 (−2.33 to 8.05)	0.241	**7.541** (−2.33 to 12.37)	**0.002**
BMI (kg/m^2^)	−0.15 (−2.33 to 0.10)	0.251	−0.13 (−2.33 to 0.13)	0.327	−0.06 (−2.33 to 0.25)	0.697	–**0.37** (−2.33 to 0.00)	**0.045**	−0.02 (−2.33 to 0.32)	0.877
Age at diagnosis (per year)	−0.24 (−2.33 to 0.03)	0.084	−0.12 (−2.33 to 0.15)	0.392	−0.24 (−2.33 to 0.09)	0.156	**−0.44** (−2.33 to −0.05)	**0.025**	0.04 (−2.33 to 0.41)	0.793
Time since diagnosis (per year)	0.10 (−2.33 to 0.50)	0.624	0.16 (−2.33 to 0.58)	0.439	0.26 (−2.33 to 0.76)	0.307	−0.28 (−2.33 to 0.28)	0.327	0.22 (−2.33 to 0.77)	0.431
Time on GFD (per year)	0.30 (−2.33 to 0.68)	0.115	0.32 (−2.33 to 0.71)	0.100	0.20 (−2.33 to 0.68)	0.386	0.42 (−2.33 to 0.96)	0.120	0.39 (−2.33 to 0.90)	0.136
CDAT score	**−1.77** (−2.33 to −1.43)	**<0.001**	–**1.83** (−2.33 to −1.49)	**<0.001**	**−1.82** (−2.33 to −1.41)	**<0.001**	–**1.69** (−2.33 to −1.22)	**<0.001**	**−1.58** (−2.33 to −1.13)	**<0.001**
Member of a CD association	−0.45 (−2.33 to 1.76)	0.688	0.27 (−2.33 to 2.54)	0.812	−1.16 (−2.33 to 1.58)	0.406	−1.15 (−2.33 to 1.97)	0.470	**3.05** (−2.33 to 6.04)	**0.046**
Has comorbidity	−0.79 (−2.33 to 1.64)	0.522	−0.13 (−2.33 to 2.37)	0.915	−0.76 (−2.33 to 2.27)	0.621	−0.25 (−2.33 to 3.20)	0.886	–**3.63** (−2.33 to −0.32)	**0.031**
No anxiety	**4.90** (−2.33 to 8.00)	**0.002**	**4.16** (−2.33 to 7.34)	**0.010**	**6.50** (−2.33 to 10.36)	**0.001**	4.36 (−2.33 to 8.75)	0.051	0.49 (−2.33 to 4.70)	0.816
No depression	**5.40** (−2.33 to 9.45)	**0.025**	**7.40** (−2.33 to 11.93)	**0.001**	**5.93** (−2.33 to 11.42)	**0.034**	1.20 (−2.33 to 7.44)	0.705	5.86 (−2.33 to 11.84)	0.054
Smoker	1.20 (−2.33 to 4.30)	0.446	2.81 (−2.33 to 5.99)	0.083	1.89 (−2.33 to 5.75)	0.335	0.36 (−2.33 to 4.75)	0.869	−3.04 (−2.33 to 1.15)	0.155

MD, mean difference; CI, confidence interval. Bold values indicate statistically significant results (*p* < 0.05).

Regarding the CD-QOL dimensions, different patterns were observed. Income gradient and age maintained positive associations with limitations and health problems, and daily exercise showed favorable effects on limitations and inadequate treatment. The absence of anxiety and depression was associated with better scores on dysphoria and limitations. Conversely, poorer adherence to a gluten-free diet maintained consistent negative associations across all four subscales.

Membership in associations was only linked to better scores on the inadequate treatment dimension. Conversely, a higher BMI and later diagnosis were associated with worse outcomes in health problems, and physical comorbidity showed a negative effect on inadequate treatment.

Time since diagnosis, years on a gluten-free diet, and smoking did not show independent associations.

## Discussion

HRQoL is modulated by biological, psychological, and social factors (1–3). An integrated analysis of these elements enables identification of areas for improvement in CD care. Within this context, the objective of this study was to evaluate how social and clinical factors influence HRQoL in adults with this condition.

The results of this study showed an association between higher quality of life scores and older age, higher income, daily exercise, and the absence of anxiety and depression. In contrast, poorer dietary adherence was associated with poorer quality of life.

The internal consistency of the CD-QOL was high overall and across most subscales; however, the “inadequate treatment” dimension showed low reliability. This may be partly explained by the small number of items in this subscale and the previously identified wording discrepancy in Item 8. Although reverse coding improved internal consistency, results for this dimension should be interpreted with caution. Similarly, the moderate-to-low internal consistency observed for the CDAT suggests that findings related to dietary adherence should be considered carefully.

Our HRQoL score was moderate and lower than that described in previous studies using the CD-QoL (8, 38). In the study by Russo et al. (38), the higher scores on this questionnaire could be explained by a more restrictive clinical selection, excluding comorbidities and complex clinical situations. Similarly, in the study by Fueyo et al. (8), this could be explained by a stronger association and better adherence to the gluten-free diet, which emerged as a main determinant of HRQoL. Furthermore, our study included patients regardless of whether they had adopted a gluten-free diet or the duration of their disease, ranging from recent diagnoses to long-term follow-up. Individuals with persistent symptoms or extraintestinal manifestations were also not excluded. This greater clinical heterogeneity offers a more comprehensive view of the situation in Spain, since controlling for variables such as whether or not a gluten-free diet was adopted (16, 28) or excluding participants with persistent symptoms (25) or complications such as dermatitis herpetiformis (15) can limit external validity by generating more homogeneous samples with better clinical profiles.

This study shows that HRQoL in celiac disease is influenced by multiple determinants, consistent with previous work. Several authors have pointed to the role of educational level (39, 40), economic status (41), and age (7, 8, 31, 42) as factors that modulate wellbeing. Regarding gender, no differences were identified in our sample, and the evidence remains heterogeneous and contradictory (4, 8, 39, 43). To rule out less visible effects and improve the ability to detect real differences, it would be necessary to increase male representation in future studies of this type.

On the other hand, the fact that physical exercise and mental health act as protective factors for HRQoL is consistent with the available evidence. The benefits associated with physical activity align with studies that document improvements in wellbeing and functioning in different clinical contexts (44–47), and our results indicate that this effect is also evident for gastrointestinal health supporting the gut-brain axis (48). Along these lines, the MOVE-C pilot study (49) already showed that a 12-week high-intensity interval training (HIIT) program, combined with lifestyle education, could improve the quality of life in patients with celiac disease. Additionally, the negative influence of anxiety and depression on HRQoL and adherence to a gluten-free diet is consistent with systematic reviews that identify these factors as key determinants in patients with celiac disease (13, 30). Likewise, mental fatigue (49) and psychological disorders (12, 50, 51) have been associated with poorer adherence and wellbeing. Overall, the data indicate that strengthening mental health by incorporating anxiety and depression screening along with psychoeducational support, as well as promoting physical activity, improves wellbeing and can optimize dietary adherence, supporting the need for multidimensional strategies in addressing celiac disease.

Regarding the diagnostic process and disease management, adherence to a gluten-free diet and membership in patient associations are confirmed as key determinants of HRQoL. The available evidence consistently indicates that early diagnosis, adequate dietary adherence, and the availability of structured dietary and social support are associated with higher levels of wellbeing in people with celiac disease (2, 8, 15, 16, 39, 50–53). In this sense, dietary adherence emerges as one of the main factors, consistent with the meta-analysis by Rustagi et al. (1), which describes significantly higher HRQoL scores among those who strictly follow the GFD. However, recent studies assessing adherence with the Celiac Diet Adherence Test indicate that a considerable proportion of adults with celiac disease still present suboptimal adherence, highlighting the need for continued nutritional support (29). From an applied perspective, these findings reinforce the need to implement comprehensive approaches that include ongoing dietary support, social and educational resources, and support from patient associations as key elements to promote a sustainable GFD and improve HRQoL.

When considering all variables together, older age, higher income, daily exercise, and absence of anxiety/depression were associated with better overall CD-QOL scores, while poor adherence was linked to worse quality of life. Furthermore, the model controlling for covariates revealed associations missed in the preliminary analysis: being female and having physical comorbidity were associated with worse outcomes in “inadequate treatment,” and a high BMI was associated with lower scores in “health problems.” These findings reflect the complexity of HRQoL, in which some effects become visible only when multiple determinants are considered together. In the Spanish context, the study by Rodríguez-Almagro et al. (31) was pioneering in applying multivariate analysis to the study of HRQoL in CD in Spain. In that study, which included 1,230 participants, the sample was also predominantly female (89.2%) and slightly younger (mean age 32.7 years), with a mean CD-QOL score of 56.3 ± 18.3. A decade later, our study shows a comparable demographic profile and a similar overall level of HRQoL, although slightly higher (59.9 ± 20.1). However, there are differences in the determinants identified in the adjusted models. Rodríguez-Almagro et al. included only sex, age, time since diagnosis, and duration of gluten-free diet in their multivariate analysis, and found that longer duration of treatment was associated with better HRQoL. In contrast, in our models neither time since diagnosis nor years on a gluten-free diet remained independently associated with HRQoL. These differences should be interpreted cautiously, as our study incorporated a broader range of socioeconomic, behavioral, and psychological variables.

When analyzing the determinants with the greatest impact, it is reasonable to suggest a positive effect of these on HRQoL. The association between higher income and better quality of life aligns with the evidence: higher income facilitates access to gluten-free products, whose high cost is a recognized barrier in the recent meta-synthesis by Kowalczuk et al. (10), and is associated with poorer emotional wellbeing and lower HRQoL when food insecurity exists (41). Beyond the direct cost of gluten-free products, these findings can also be interpreted in terms of purchasing power and economic accessibility. Individuals with lower income may face greater difficulty sustaining a strict gluten-free diet due not only to higher relative food costs, but also to reduced flexibility in food choices, limited access to specialized products, and potential constraints in accessing professional dietary support. In this context, the financial burden of a gluten-free diet may disproportionately affect lower-income groups, amplifying inequalities in health-related quality. Age, for its part, already showed a consistent effect in the multivariate analysis by Rodríguez-Almagro et al. (31) and could be linked to greater experience in managing the disease, as suggested by Castilhos et al. (42).

In the analysis by dimensions, dysphoria was the domain with the highest score. After multivariable analysis, mental health emerged as its main determinant, and better adherence to a gluten-free diet showed an additional favorable effect. These findings are consistent with the literature, which describes anxiety and depression as being associated with poorer quality of life and a greater emotional burden in celiac disease. Möller et al. (13) reported that greater concern about food safety and handling is related to lower scores on the CD-QOL, and that the presence of a “probable mental disorder” (HADS ≥ 11) increases the likelihood of obtaining a low mental component score on the SF-36 quality of life questionnaire by 11.9 times. Furthermore, the review by Xhakollari et al. (30) shows that anxiety and depression negatively influence adherence to a gluten-free diet, reinforcing the interdependence between mental health, adherence, and quality of life. Taken together, the data reinforce the central role of mental health and dietary adherence in the emotional perception of the impact of the disease.

The dimension with the lowest score was “limitations,” reflecting a significant impact on daily activities and the perception of restrictions associated with the disease. In this domain, the most influential factors were higher income, daily exercise, and the absence of anxiety and depression, all associated with better outcomes. In practice, equity measures can mitigate inequalities arising from the cost of gluten-free products, consistent with previous studies on economic barriers and financial burden (6, 10). Furthermore, the review by Postl-Königová M et al. (12) shows that programs combining physical activity, education, and coping skills training generate more consistent improvements in functioning, symptoms, and perceived limitations. Taken together, the data support strategies focused on improving economic accessibility, strengthening health education, and promoting healthy lifestyles. These interventions appear promising for reducing perceived limitations and optimizing this dimension of HRQoL. In particular, people with lower socioeconomic status could benefit from more intensive interventions in dietary education, practical training, and community support.

In the “health problems” dimension, the income gradient again predominated, suggesting that economic inequalities also modulate the perceived clinical burden. Older age showed an additional positive effect. Conversely, a higher BMI, older age at diagnosis, and suboptimal adherence were associated with worse scores. From a clinical perspective, these findings reinforce the need to adjust nutritional management, promote early diagnosis pathways, and maintain regular follow-up to consolidate adherence. The literature has described how expanded health education improves adaptation (12), how establishing a dietary change plan and the passage of time fosters experience and self-management (3, 16, 31, 39), and how adequate adherence is a key determinant of wellbeing (1, 2, 27, 54). The observed income gradient confirms that CD is not only a clinical process but also a condition shaped by structural inequalities that influence wellbeing.

Finally, in the “inadequate treatment” dimension, daily exercise and membership in an association were associated with better scores. However, association membership was not significantly associated with the psychosocial domains of dysphoria or limitations. This may suggest that the support provided by these organizations is mainly focused on practical information and disease management rather than structured emotional support, although this aspect was not directly assessed in our study. Conversely, physical comorbidity, poor adherence, and female sex showed a negative impact. It is worth highlighting the modifiable factors: participation in associations provides psychoeducational support, reinforces adherence, and promotes healthy habits, something already described in previous studies (28, 39). These findings highlight that improving adherence, reducing comorbidity, and promoting active participation in support groups not only optimize the treatment experience but also strengthen patients' self-management capacity.

In the adjusted models, neither the number of years since diagnosis nor the time spent on a gluten-free diet were significant. This suggests that it is not the time itself, but rather the accompanying conditions: resources for a safe gluten-free diet, psychosocial support, coping strategies, sustained adherence, and healthy habits (especially physical activity). At the same time, this process may not be strictly linear. Although longer disease duration may promote adaptation and self-management, long-term adherence to a strict gluten-free diet can also impose a sustained psychological and social burden. For some individuals, this may lead to treatment fatigue or “coeliac burnout”, characterized by persistent vigilance, dietary restrictions, and social limitations. This ongoing burden may counterbalance the benefits of experience and contribute to a plateau in HRQoL over time, as suggested by previous studies highlighting the chronic psychosocial impact of maintaining a gluten-free diet (55, 56). In practice, HRQoL improves less by “accumulating months” and more by how that period is experienced: with dietary safety, stable mental health, an active lifestyle, and good adherence.

This study, to our knowledge, is the first in Spain to analyze a large sample of adults with celiac disease using an integrated approach that incorporates a broad range of social, health, and clinical factors. The combined use of bivariate and multivariate analyses allows for more precise identification of independent determinants of quality of life. Furthermore, the application of validated instruments (CD-QOL and CDAT) and a multidimensional assessment based on global scores and subscales offers a more robust and representative estimate of clinical experience in celiac disease. The breadth of variables included provides a more comprehensive perspective than that available in previous studies.

Limitations include low male participation, which may reduce generalizability by gender; the cross-sectional design, which prevents establishing causality; and the use of self-reports, which are subject to measurement bias. Additionally, physical activity was assessed using a non-validated, self-reported frequency measure without information on duration or intensity, which may limit the accuracy of this variable. In particular, anxiety and depression were based on self-reported prior diagnosis rather than assessed using validated psychometric scales, which may underestimate current psychological distress. The study did not include a detailed assessment of specific gastrointestinal or extraintestinal symptoms, which may limit the ability to explore their independent contribution to HRQoL.

Future studies should incorporate more balanced sampling strategies, longitudinal designs, and objective measures of adherence and clinical variables. In addition, uncollected information (such as histological severity or functional gastrointestinal disorders) could improve the explanatory model. Information on access to professional dietitian support was not collected in our study, although such support could influence dietary adherence and HRQoL. Moreover, the online survey format may have limited participation among some older adults or individuals with lower digital literacy, which could affect the representativeness of the sample. Recruitment through patient associations may have introduced a potential selection bias, as individuals connected to these organizations may have higher health literacy, greater engagement with disease management, and potentially better adherence to a gluten-free diet than the general celiac population. However, approximately half of the participants in our sample were not members of patient associations, which may partially mitigate this bias. Nevertheless, the findings should be interpreted with caution when generalizing the results to all adults with celiac disease in Spain.

## Conclusions

In this national sample of adults with celiac disease, HRQoL was modulated by a broad set of personal, health, and lifestyle factors, along with characteristics specific to celiac disease.

In multivariate models, older age, higher income level, daily exercise, and the absence of anxiety and depression were independently associated with better overall CD-QOL scores. Adherence to a gluten-free diet emerged as the most relevant clinical determinant, with consistent negative associations across all dimensions when adherence was suboptimal.

In contrast, variables such as time since diagnosis or years on a gluten-free diet did not show independent associations, indicating that disease duration alone does not explain the variability in quality of life. Additionally, BMI, age at diagnosis, and physical comorbidity showed specific effects on certain dimensions of the CD-QOL.

These findings highlight that quality of life in celiac disease is primarily influenced by socioeconomic, behavioral, and psychological factors, rather than simply the passage of time since diagnosis. Consequently, this supports the design of integrated interventions that combine dietary education with strategies to improve adherence, promote physical activity, screen for and implement interventions for anxiety and depression, and implement equity measures to reduce the economic burden associated with gluten-free products. This multidimensional approach can contribute to improving wellbeing and reduce inequalities in this population.

## Data Availability

The raw data supporting the conclusions of this article will be made available by the authors, without undue reservation.
